# Arginine methylation expands the regulatory mechanisms and extends the genomic landscape under E2F control

**DOI:** 10.1126/sciadv.aaw4640

**Published:** 2019-06-26

**Authors:** Alice Poppy Roworth, Simon Mark Carr, Geng Liu, Wojciech Barczak, Rebecca Louise Miller, Shonagh Munro, Alexander Kanapin, Anastasia Samsonova, Nicholas B. La Thangue

**Affiliations:** 1 Laboratory of Cancer Biology, Department of Oncology, Medical Sciences Division, University of Oxford, Old Road Campus Research Building, Old Road Campus, Roosevelt Drive, Oxford OX3 7DQ, UK.; 2 Institute of Translational Biomedicine, St. Petersburg University, St. Petersburg 199034, Russia.

## Abstract

E2F is a family of master transcription regulators involved in mediating diverse cell fates. Here, we show that residue-specific arginine methylation (meR) by PRMT5 enables E2F1 to regulate many genes at the level of alternative RNA splicing, rather than through its classical transcription-based mechanism. The p100/TSN tudor domain protein reads the meR mark on chromatin-bound E2F1, allowing snRNA components of the splicing machinery to assemble with E2F1. A large set of RNAs including spliced variants associate with E2F1 by virtue of the methyl mark. By focusing on the deSUMOylase SENP7 gene, which we identified as an E2F target gene, we establish that alternative splicing is functionally important for E2F1 activity. Our results reveal an unexpected consequence of arginine methylation, where reader-writer interplay widens the mechanism of control by E2F1, from transcription factor to regulator of alternative RNA splicing, thereby extending the genomic landscape under E2F1 control.

## Teaser

Arginine methylation widens the mechanism of control by E2F1 from a transcription factor to a regulator of alternative RNA splicing.

## INTRODUCTION

E2F is a family of master transcription regulators involved in mediating diverse cell fates, which frequently becomes deregulated in cancer. The retinoblastoma protein (pRb)–E2F pathway is a central player in the control of cell cycle progression in diverse cell types and its deregulation of primary importance in proliferative disease such as cancer, where aberrant pRb activity occurs through a variety of oncogenic mechanisms (*1*). In the classical view, cyclin-dependent kinases, which peak during the G1 phase phosphorylate pRb, cause the release of E2F from the pRb/E2F complex, enabling E2F to transcriptionally activate target genes required for cell cycle progression (*2*–*5*). E2F1 is one of the most important physiological targets for pRb, and the physical interaction between pRb and E2F1 facilitates transcriptional repression and cell cycle arrest (*1*, *2*). However, E2F1 can foster other biological outcomes such as the induction of apoptosis (*6*–*8*). Understanding the molecular mechanisms responsible for regulating the diverse biological outcomes of E2F1 activity remains a central question in E2F biology which, further, has direct relevance to its pathological role in cancer.

Methylation of arginine side chains is becoming increasingly recognized as an important protein modification involved with diverse pathways of control (*9*, *10*). In previous studies, we identified a small R-rich motif in E2F1 as a target for arginine methylation (*11*, *12*) and uncovered a remarkable relationship between methylation by protein arginine methyltransferase 5 [PRMT5 (symR)] and PRMT1 (asymR) in channeling E2F1 through its distinct biological pathways (*11*, *12*); thus, PRMT5-dependent methylation prompts cell growth, in contrast to methylation by PRMT1 that facilitates apoptosis (*11*, *12*). The symR E2F1 mark is read by the tudor domain protein, p100/tudor staphylococcal nuclease (TSN) (*12*), which exists as a chromatin-bound symR E2F1 complex on E2F target genes (*12*, *13*). Furthermore, PRMT5-dependent methylation is uniquely relevant to E2F1 among the E2F family (*11*, *12*), suggesting that the meR mark is fundamental in the control of E2F1 activity.

Here, we show that methylation by PRMT5 enables E2F1 to regulate a diverse group of genes at the level of alternative RNA splicing, rather than through the classical transcription-based mechanism widely ascribed to E2F1. The impact of E2F1 on alternative RNA splicing requires the tudor domain protein p100/TSN to read the meR mark, allowing components of the splicing machinery such as small nuclear RNA (snRNA) to associate with the p100/TSN-E2F1 complex. Consistent with its role in RNA splicing, a large group of RNAs, including spliced intermediates, bind to the E2F1 complex. Most genes subject to alternative splicing are poor transcription targets for E2F1. We identified *SENP7* as a previously unidentified E2F target gene subjected to alternative RNA splicing control by E2F1. At the functional level, SENP7 (SUMO1/sentrin specific peptidase 7) protein influenced E2F target gene activity through regulating chromatin SUMOylation and heterochromatin protein 1 (HP1) binding. Our results reveal an unexpected role for E2F1 in regulating the alternative RNA splicing machinery, which occurs through a meR mark–dependent reader-writer interplay, enabling E2F1 to broaden its influence to genes that otherwise are poor transcription targets. The methyl mark, therefore, confers a new mechanism of control and extends the genomic landscape under E2F1 control.

## RESULTS

### meR marks on E2F1 confer genome-wide effects

**Fig. 1:**
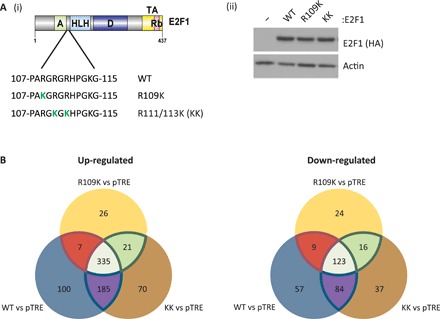
meR marks on E2F1 confer genome-wide effects. (**A**) Schematic representation of E2F1, highlighting the region of the protein targeted by PRMT1 and PRMT5. The arginine methylation-defective E2F1 derivatives [R109K and R111/113 K (KK)] used to generate U2OS stable cell lines for RNA-seq analysis are also indicated (i). An immunoblot displaying E2F1 protein expression in U2OS stable cells after 24 hours of doxycycline (1 μg/ml) treatment is also included (ii). See also fig. S1 (A to D). (**B**) Venn diagrams showing the crossover of genes up- or down-regulated over twofold (adjusted *P* value threshold < 0.01) in each cell line condition with respect to the pTRE empty vector cell line, filtered for genes containing an E2F1 motif in their proximal promoter region (−900 to +100). These data were generated from three independent biological samples.

To clarify the role of the meR mark in regulating E2F1 activity, we developed a panel of Tet-On inducible cell lines (Fig. 1A). Each cell line expressed wild-type (WT) E2F1 or its derivative KK (with mutated symR sites at R111 and R113) previously established to be defective in PRMT5 methylation and to exert apoptosis more efficiently than WT E2F1 (*12*). For comparison, we prepared a cell line expressing R109K (with a mutated asymR site at R109), which cannot be methylated by PRMT1 but retains the PRMT5 symR sites; the R109K derivative is endowed with a greater proliferation activity relative to WT E2F1 (*12*). The induced WT, KK, and R109K E2F1 proteins behaved as expected; upon expression, each ectopic protein underwent nuclear accumulation and by chromatin immunoprecipitation (ChIP) localized to the promoter region of E2F target genes (fig. S1, A and B) and exhibited similar binding and cellular activities as described previously (fig. S1, C and D) (*12*).

We used RNA sequencing (RNA-seq) to assess the global transcript profile in each stable cell line. Mining the RNA-seq dataset for transcripts regulated twofold or more upon E2F1 expression (compared to the empty pTRE vector cell line) identified a large number, the majority (around 50% for each cell line) being derived from E2F target genes (fig. S1E and table S1), where an E2F target gene was defined by the presence of one or more E2F binding site consensus motifs in the proximal promoter region (−900 to +100) (*14*). For the WT E2F1 expression condition, 900 E2F target gene transcripts whose expression was affected by more than twofold were identified (Fig. 1B and fig. S1E), a figure broadly in line with previous reports on E2F transcription targets (*15*).

Within the population of twofold regulated transcripts, the majority were up-regulated, although a substantial proportion were also down-regulated (70% compared to 30%, respectively; fig. S1E). Further, although a high proportion of up- and down-regulated transcripts were shared between the WT and KK E2F1 cell lines (80.7 and 83.5%, respectively) (Fig. 1B), some of the transcripts were nonoverlapping and, therefore, independently regulated by either WT or KK E2F1 expression. A total of 17.1 and 14.9% of the transcripts were differentially up-regulated by either WT or KK, respectively, and conversely, 24.2 and 20.4% were differentially down-regulated (Fig. 1B).

We performed a similar analysis on the R109K expression condition. In contrast to WT or KK E2F1, the R109K derivative was less able to influence transcription (Fig. 1B and fig. S1E); notably, R109K was about 60% as efficient as WT E2F1 in regulating transcription. Further, 93% of the transcripts up-regulated by R109K were shared with either WT or KK E2F1, with only 26 unique transcripts detected in the R109K expression condition (Fig. 1B). We observed a similar pattern when down-regulated transcripts were analyzed; again, 86% of the transcripts down-regulated by R109K were shared with WT and KK E2F1, with 24 unique transcripts apparent (Fig. 1B and fig. S1E). Thus, R109K, which retains intact residues R111 and R113 methylated by PRMT5, is less able to regulate transcription than its WT and KK counterparts.

We assessed the gene sets that were present in the RNA-seq by gene set analysis. There were a number of shared gene sets enriched in each condition, including E2F targets (as expected), whereas gene sets connected with the epithelial-mesenchymal transition and hypoxia were generally down-regulated in each condition (fig. S2A).

It was important to validate the results from the RNA-seq. We therefore measured the expression of a number of E2F target candidate genes identified in the RNA-seq dataset, where there was evidence for differential expression patterns. For example, *LRRC4*, *ETV1*, and *FGF4* transcripts were expressed at high levels in the KK cell line, with reduced expression in the R109K cell line, and a similar pattern of expression was evident when transcription from each gene was individually measured in each cell line (fig. S1F). Conversely, at the global level, *KCNIP* showed higher expression in R109K compared to KK, and a similar expression pattern was apparent when gene expression was individually measured (fig. S1F). Moreover, we confirmed that the expression of each candidate gene was dependent on E2F1, as silencing endogenous E2F1 with siRNA caused reduced expression of each gene (fig. S1G).

### E2F1 permits alternative RNA splicing of E2F target genes

**Fig. 2:**
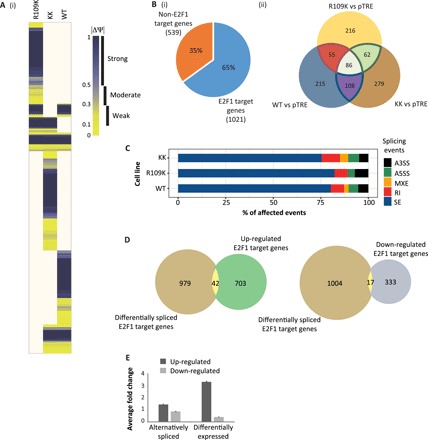
E2F1 affects alternative splicing of E2F target genes. (**A**) A heatmap displaying absolute values of ΔΨ (percent spliced in) for each cell line, corresponding to statistically significant alternative splicing event changes to E2F1 target genes (as determined by the presence of ChIP-seq peaks in their promoter and gene regions, retrieved from ENCODE data) with respect to the pTRE empty vector cell line, derived by analyzing the RNA-seq data with rMATS algorithm. Yellow color represents the lowest difference, and blue color represents the highest. Ivory blocks correspond to nonsignificant changes in splicing patterns (FDR > 0.01). See also table S2 and fig. S3. (**B**) Pie chart showing the percentage of genes identified in the rMATS splicing analysis, which are E2F1 target genes (as determined by the presence of ChIP-seq peaks in their promoter and gene regions, retrieved from ENCODE data) (i). The Venn diagram demonstrates the overlap of E2F1 target genes affected by alternative splicing events (FDR < 0.01) in each cell line (ii). These data were generated from three independent biological samples. (**C**) Bar chart displaying the statistically significant alternative splicing events to E2F target genes for each cell line, as compared to the pTRE vector control. The percentage of these alternative splicing changes corresponding to different types of splicing event is displayed in different colors. SE, skipped/cassette exon; RI, retained intron; MXE, mutually exclusive exons; A5SS, alternative 5′ splice site; A3SS, alternative 3′ splice site. See also fig. S2B. (**D**) Venn diagrams showing overlap between E2F1 target genes identified in the differential expression analysis as being up- or down-regulated (regulated greater than twofold; Fig. 1B) and those identified as being differentially or alternatively spliced [(A) and table S2]. These data were generated from three independent biological samples. (**E**) Bar chart representing the average fold change in expression of differentially expressed E2F1 target genes (regulated greater than twofold), compared with the expression of those E2F1 target genes where alternative splicing occurred. Only 389 genes from the alternative splicing analysis met the significance threshold for differential expression (*P* < 0.01). The remaining 632 spliced genes had expression levels that were not significant from the pTRE empty vector cell line (*P* > 0.01) and were therefore assigned an arbitrary value of 1 for this analysis.

It is noteworthy that the R109K derivative exhibits a reduced ability to affect transcription (Fig. 1B and fig. S1E). Because p100/TSN interacts with the splicing machinery (*16*), and R109K binds to p100/TSN through PRMT5-dependent methylation of residues R111 and R113 (*12*), we reasoned that p100/TSN may confer on E2F1 the ability to control RNA splicing. We therefore mined each RNA-seq dataset for evidence of alternative RNA splicing using the rMATS algorithm (Fig. 2A) (*17*). Of great interest was the fact that a large number of transcripts derived from 1560 genes were present in the dataset, where 1021 (namely, 65%; Fig. 2B), identified as E2F target genes, exhibited alternative splicing effects dependent on E2F1 expression.

We observed alternative splicing events in E2F gene transcripts, which included skipped exons, alternative 3′ (A3SS) or 5′ (A5SS) splice sites, mutually exclusive exons, and retained introns (Fig. 2C and fig. S2B). Some transcripts were subject to different alternative splicing events (table S2). Notably, although defined as E2F target genes by the presence of canonical E2F DNA binding motifs and cross-referencing to ChIP-seq datasets in the encyclopedia of DNA elements (ENCODE) (ENCODE project consortium, 2012), we found upon mining the RNA-seq data that the vast majority of alternatively spliced E2F transcripts were modest transcription targets for E2F1 (transcriptionally regulated less than twofold upon the expression of E2F1; Fig. 2, D and E). Only 42 genes in the transcriptionally up-regulated E2F target gene group, and 17 genes in the down-regulated group, were shared with the alternatively spliced set (less than 3% overlap between the two sets of genes); most of the E2F1-dependent alternative RNA splicing thus occurred on genes that are poor transcription targets for E2F1 (Fig. 2, D and E). The results highlight two categories of E2F target genes, one made up of genes, which are good transcription targets (regulated greater than twofold by E2F1), and the other composed of genes principally regulated through alternative splicing, which, generally, are poor transcription targets.

When each set of alternatively spliced transcripts derived from E2F target genes was compared under the WT, KK, and R109K E2F1 expression conditions, qualitative and quantitative differences in the alternatively spliced RNA were apparent, with events that were both shared and unique (Fig. 2A). WT and KK cell lines shared 41 and 36% of the alternatively spliced genes, while WT and R109K shared 30 and 34%, and KK and R109K shared 28 and 34% (Fig. 2B, ii), highlighting the fact that each E2F1 derivative affects alternative splicing of an overlapping set of RNAs. Further, R109K caused the strongest splicing effect (by rMATS analysis) contrasting with KK, which was least efficient (fig. S2B). This situation contrasted with the transcription analysis of the RNA-seq data (Fig. 1B), where R109K was less effective than the WT and KK derivatives in causing differential gene expression.

We performed gene ontology (GO) analysis on the E2F gene sets from which the alternatively spliced transcripts were derived (fig. S3). Although there was considerable overlap in the GO terms enriched in each condition, such as cellular processes linked to cell cycle, there were a number of marked differences. For example, DNA damage–related terms were prevalent under KK expression conditions, while catabolic and biosynthetic terms were enriched upon R109K expression.

We also studied the expression level of a variety of E2F target genes connected with splicing, many encoding components of the splicing machinery (table S3). From an analysis of the RNA-seq data, none of the genes were expressed at a notably different level under the WT, KK, or R109K expression conditions (table S3). The increased level of alternative splicing identified by rMATS, therefore, cannot be easily attributed to coincident changes in the expression of splicing components.

### Chromatin-associated E2F1 binds to components of the splicing machinery

**Fig. 3:**
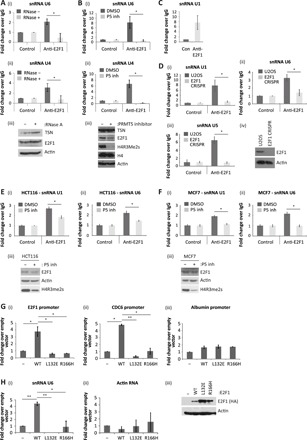
E2F1 interacts with components of the splicing machinery. (**A**) U2OS cells were lysed in RIP lysis buffer, containing ribonuclease A (RNase A; 20 μg/ml) where indicated. Cell extracts were immunoprecipitated with E2F1 antibody, and coimmunoprecipitated RNA was reverse-transcribed before quantitative polymerase chain reaction (qPCR) analysis with primers against U6 (i) and U4 (ii) snRNAs as indicated. Input protein levels were determined by immunoblot (iii). *n* = 2. (**B**) U2OS cells were treated with 5 μM PRMT5 inhibitor (P5 inh), as indicated, before performing an anti-E2F1 RIP. Coimmunoprecipitated U6 (i) and U4 (ii) snRNAs were identified with specific primers by quantitative reverse transcription PCR (qRT-PCR). Input protein levels were determined by immunoblot (iii). *n* = 3. (**C**) An anti-E2F1 RIP was performed on U2OS cells, and coimmunoprecipitated U1 snRNA was detected by qRT-PCR. *n* = 2. (**D**) An anti-E2F1 RIP was performed on extracts prepared from U2OS or U2OS E2F1 CRISPR cell lines as indicated. Immunoprecipitated RNA was analyzed by qRT-PCR using primers specific to U1 (i), U6 (ii), or U5 (iii) snRNAs. Input protein levels are also displayed (iv). *n* = 2. (**E**) HCT116 cells were treated with 5 μM PRMT5 inhibitor, where indicated, before performing an anti-E2F1 RIP. Coimmunoprecipitated U1 (i) and U6 (ii) snRNA were detected by qRT-PCR. Input protein levels are also displayed (iii). *n* = 2. (**F**) As described above, although the experiment was performed in MCF7 cells. (**G**) U2OS cells were transfected with 1 μg of plasmid encoding WT E2F1, DNA binding domain mutant constructs (L132E and R166H) or empty vector (−) as indicated. Forty-eight hours later, cell extracts were used for ChIP analysis with the anti–hemagglutinin (HA) antibody. Immunoprecipitated chromatin was analyzed by qPCR using primers targeting the indicated promoters, where albumin served as the non-E2F target gene control (i to iii). Input protein levels are shown in (H). *n* = 2. See also fig. S4B. (**H**) U2OS cells were transfected as above. Forty-eight hours later, cell extracts were used for RIP analysis with anti-HA antibody. Immunoprecipitated RNA was analyzed by qRT-PCR using primers specific to U6 snRNA (i) or actin RNA (ii). Input protein levels were determined by immunoblot (iii). *n* = 3.

We reasoned that the impact of E2F1 on alternative RNA splicing could be mediated by meR E2F1 interacting with components of the splicing machinery, since the meR reader protein p100/TSN functions in spliceosome assembly and enhances splicing activity (*16*). We therefore addressed whether snRNAs, essential components of the spliceosome (*18*), could associate with E2F1. By RNA immunoprecipitation (RIP), we found that U1, U4, U5, and U6 snRNAs associate with E2F1 in a variety of cell types, including U2OS, HCT116, and MCF7 cells (Fig. 3, A to F). Significantly, the interaction of snRNA with E2F1 was dependent on p100/TSN and PRMT5 activity, as it was reduced in cells treated with p100/TSN siRNA (fig. S4A) and absent in cells treated with the PRMT5 inhibitor EPZ015666 (Fig. 3, B, E, and F) (*19*). To assess whether snRNA binding to E2F1 required an intact DNA binding domain and therefore was likely to occur with chromatin-associated E2F1, we prepared E2F1 derivatives with compromised DNA binding activity (Fig. 3G). By ChIP, neither L132E nor R166H bound to chromatin relative to WT E2F1 (Fig. 3G), although each mutant derivative could undergo nuclear accumulation (fig. S4B). Significantly, upon RIP analysis with L132E or R166H, the level of U6 snRNA was reduced, in contrast to the WT E2F1 RIP where U6 was clearly detectable (Fig. 3H), arguing that the interaction with snRNA occurs with chromatin-bound E2F1.

### E2F1 interacts with a diverse set of alternatively spliced transcripts

Having established that arginine methylation and its reader p100/TSN enable E2F1 to influence alternative splicing (Fig. 2) and further allow binding to snRNA, we went on to explore whether any additional RNA species could associate with p100/TSN-E2F1 using RIP sequencing (RIP-seq). We performed the E2F1 RIP-seq analysis in the presence and absence of p100/TSN to characterize the population of RNA that bound to E2F1 in a meR-p100/TSN–dependent fashion. We observed a large set of RNAs, 384 in total, in the E2F1 RIP-seq that were dependent on the presence of p100/TSN (table S4). Some of the p100/TSN-dependent RNAs identified in the E2F1 RIP-seq were highlighted to be alternatively spliced RNAs in the rMATS splicing analysis of the RNA-seq dataset (Fig. 2A and table S5). For example, the lysine acetyl-transferase 2B (*KAT2B*) (ΔΨ = 0.171 to 0.232), the lysine methyl-transferase SET domain containing 2 (*SETD2*) (ΔΨ = −0.888), and max gene associated protein (*MGA*) (ΔΨ = 0.436) were identified as alternatively spliced transcripts by rMATS (table S2).

**Fig. 4:**
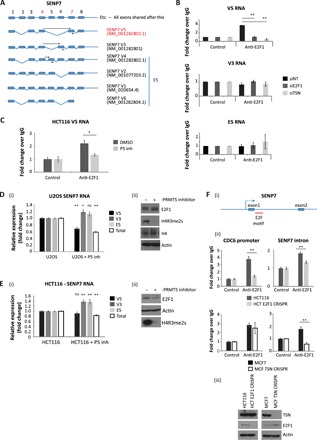
p100/TSN enables E2F1 to interact with alternatively spliced transcripts. (**A**) Schematic representation of exon structure for the SENP7 gene. Each alternatively spliced transcript expressed from this gene is displayed, with primer binding sites used to detect specific transcript variants in subsequent experiments indicated with black arrows. Note that forward primers were designed to span exon junctions. Mining of the RIP-seq dataset for exon spanning peaks identified reads around exons 4 and 7 (indicated by the red numbering), which occurs in SENP7 transcript V5 (highlighted in red text). (**B**) Anti-E2F1 RIP with U2OS cells treated with siRNA against E2F1, TSN, or nontargeting (NT) control, as indicated, for 72 hours. Cells were then immunoprecipitated with E2F1 antibody, and coimmunoprecipitated RNA was reverse-transcribed before qPCR analysis with primers against specific SENP7 transcript variants as indicated. *n* = 3. (**C**) HCT116 cells were treated with 5 μM PRMT5 inhibitor, where indicated, before performing an anti-E2F1 RIP. Coimmunoprecipitated SENP7 V5 transcripts were analyzed by qRT-PCR. Input protein levels are the same as those displayed in Fig. 3E. *n* = 2. DMSO, dimethyl sulfoxide. (**D**) U2OS cells were treated for 72 hours with 5 μM PRMT5 inhibitor. RNA was then isolated from cells and analyzed by qRT-PCR using primers targeting specific SENP7 transcript variants or total SENP7 RNA. Average (mean) fold change of each RNA species as compared to untreated U2OS cells was calculated and displayed with SE. Statistical analysis for each condition compared to untreated U2OS cells is also displayed over each bar (i). An immunoblot to demonstrate input protein levels is also included (ii). *n* = 3. (**E**) As described above, although the experiment was performed in HCT116 cells. *n* = 4. ns, not significant. (**F**) Examination of the promoter region of the SENP7 gene (–2 to +1 kb) identified an E2F1 DNA binding motif within +450 bp of the transcription start site, lying within the first intron (E2F1 motif marked in red) (i). An E2F1 ChIP was performed in the HCT116 E2F1 CRISPR and MCF7 TSN CRISPR cell lines. Immunoprecipitated chromatin was analyzed using primers spanning the identified E2F DNA binding motif in SENP7 or against the known E2F motif in the promoter sequence of CDC6 (ii). An immunoblot is included to demonstrate input protein levels (iii). *n* = 3

**Fig. 5:**
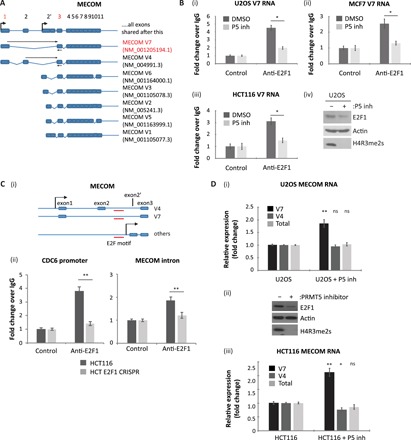
E2F1 also interacts with alternatively spliced transcripts from the MECOM gene. (**A**) Schematic representation of exon structure for the MECOM gene. Each alternatively spliced transcript expressed from this gene is displayed, with primer binding sites used to detect specific transcript variants in subsequent experiments indicated with black arrows. Note that forward primers were designed to span exon junctions. Mining of the RIP-seq dataset for exon spanning peaks identified reads spanning exons 1 and 3 (indicated by the red numbering), which occurs in MECOM transcript V7 (highlighted in red text). (**B**) U2OS (i), MCF7 (ii), or HCT116 cells (iii) were treated with 5 μM PRMT5 inhibitor as indicated. An anti-E2F1 RIP was then performed, and coimmunoprecipitated MECOM transcript variant V7 was analyzed by qRT-PCR using specific primers. Input protein levels for the U2OS experiment are also included (iv), while the input protein levels for HCT116 and MCF7 cells are the same as those displayed in Fig. 3 (E and F). *n* = 2. (**C**) Examination of the promoter region of the MECOM gene identified an E2F1 DNA binding motif lying within the first intron of V7 or the second intron of V4 (E2F1 motif marked in red) (i). An E2F1 ChIP was performed in HCT116 or HCT116 E2F1 CRISPR cell lines. Immunoprecipitated chromatin was analyzed using primers spanning the identified E2F DNA binding motif in MECOM or against the known E2F motif in the promoter sequence of CDC6 (ii). Input protein levels are the same as those displayed in Fig. 4F. *n* = 3. (**D**) U2OS cells (i) or HCT116 cells (iii) were treated with 5 μM PRMT5 inhibitor, where indicated. RNA was then isolated from cells and analyzed by qRT-PCR using primers targeting specific MECOM transcript variants or total MECOM RNA. Average (mean) fold change of each RNA species as compared to untreated U2OS/HCT116 cells was calculated and displayed with SE. Statistical analysis for each condition compared to untreated cells is also displayed over each bar. Input protein levels for U2OS cells are also displayed (ii), while the input protein levels for HCT116 cells are the same as those displayed in Fig. 4E. *n* = 4.

We further mined the E2F1 RIP-seq dataset to identify peak sequencing reads that span exon junctions across the different RNAs, which were then related to genomic organization of the parent gene, enabling us to identify spliced RNA variants. We identified a subgroup of the 384 RNA species where the sequencing reads spanned 27 exon junctions, which correspond to 26 different transcripts derived from 18 genes (table S6). For example, multiple alternatively spliced transcripts derived from *SENP7*, MDS1 and EVI1 complex locus (*MECOM*), *P3H2*, and *SPG21* genes were identified in the E2F1 RIP-seq (Figs. 4A and 5A and fig. S4, C and D) with similar alternative splicing events apparent in the RNA-seq data (*SENP7* and *MECOM* shown as Sashimi plots in fig. S4E).

We chose *SENP7* and *MECOM* as the representative examples and characterized them in greater detail. SENP7 is a deSUMOylase that is involved with the control of protein stability, chromatin, and transcription (*20*–*22*). The *SENP7* alternatively spliced RNA variant identified in the RIP-seq, V5, spanned exon junctions 4 and 7, and thus lacked exons 5 and 6 (Fig. 4A). Consistent with the RIP-seq results, the V5 RNA variant was detected in the E2F1 RIP, contrasting with the other *SENP7* RNA spliced variants (Fig. 4B). In addition, the presence of *SENP7* V5 variant in the RIP was dependent on E2F1 and p100/TSN, as it was absent upon silencing either E2F1 or p100/TSN protein (Fig. 4B). Further, inhibiting PRMT5 activity with EPZ015666 reduced the interaction between E2F1 and V5 RNA in cells (Fig. 4C), which also coincided with a lower level of the RNA variant (in contrast the other *SENP7* RNA variants increased) in cells (U2OS and HCT116) treated with EPZ015666 (Fig. 4, D and E).

To confirm that *SENP7* is a target gene for E2F1, we inspected the genomic DNA sequence around the promoter region (−2 to +1 kb) and identified an intronic E2F DNA binding site motif within 450 base pairs (bp) of the transcription start site, after the first exon (Fig. 4F). By ChIP, this region of the *SENP7* gene was capable of binding E2F1 (Fig. 4F). Moreover, using CRISPR cell lines, which lacked E2F1 or p100/TSN, we confirmed that the *SENP7* ChIP activity is dependent on E2F1 and is influenced by the presence of p100/TSN (Fig. 4F). These results highlight a role for PRMT5, E2F1, and p100/TSN in directing alternative splicing of *SENP7*.

We performed a similar analysis of *MECOM*, which encodes a zinc finger transcription factor involved with different signaling pathways (*23*). The major *MECOM* RNA species identified in the RIP-seq was the V7 spliced variant (Fig. 5A). We subsequently verified binding of the V7 RNA variant to E2F1 in diverse cell types (U2OS, HCT116, and MCF7) and the dependency on PRMT5 activity for the RNA interaction with E2F1 (Fig. 5B). By ChIP, we identified an E2F binding site within the first intron of the V7 transcript variant (Fig. 5C), and alternative splicing of *MECOM* RNA in cells was altered upon PRMT5 inhibition (Fig. 5D). Most significantly, we examined whether the *MECOM* V7 was present in human cancer by exploring RNA-seq datasets available in The Cancer Genome Atlas (TCGA) (https://cancergenome.nih.gov/) and thereafter whether there was any correlation with E2F1 and PRMT5 expression. The *MECOM* V7 transcript variant was present at increased levels in cervical, colon, and ovarian cancer compared to the normal tissue control where, importantly, its level coincided with the expression of E2F1 and PRMT5 (fig. S5). These results highlight the role of PRMT5 and p100/TSN-E2F1 in regulating alternative splicing of *SENP7* and *MECOM* RNA and further suggest that it is relevant to clinical disease.

### Biological consequence of alternative splicing for E2F1 activity

**Fig. 6:**
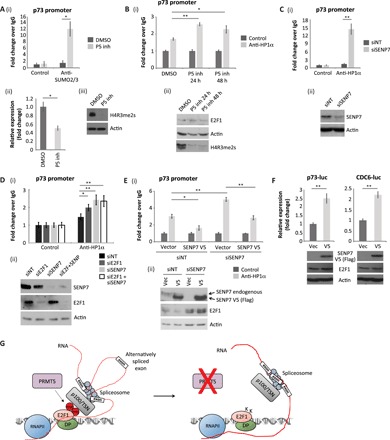
Biological consequence of SENP7 alternative splicing for E2F1 activity. (**A**) U2OS cells were treated with 5 μM PRMT5 inhibitor for 72 hours, where indicated, before ChIP analysis with anti-SUMO2/3–specific or control antibodies. Immunoprecipitated chromatin was analyzed using primers specific for the E2F site in the p73 promoter (i). An RT-PCR was also performed to monitor the levels of p73 transcripts in the cell (ii). An immunoblot for H4R3me2s is included to demonstrate the activity of the PRMT5 inhibitor (iii). *n* = 3. See also fig. S4 (F and G). (**B**) As described above, although cells were treated with the PRMT5 inhibitor for 24 or 48 hours as indicated. ChIP analysis was performed with anti-HP1α–specific or control antibodies (i). An immunoblot for H4R3me2s is included to demonstrate the activity of the PRMT5 inhibitor (ii). *n* = 2. (**C**) U2OS cells were transfected with SENP7 siRNA or nontargeting siRNA (siNT) for 96 hours as indicated. Cells were then prepared for ChIP analysis as described above (i). An immunoblot is included to demonstrate input protein levels (ii). *n* = 4. (**D**) ChIP analysis as described above, although U2OS cells were transfected with siRNA targeting E2F1, SENP7, or a combination of the two (siE2F1 + siSENP7). *n* = 3. (**E**) U2OS cells were transfected with siRNA targeting SENP7 or nontargeting siRNA for 96 hours, as indicated. Cells were subsequently transfected for 48 hours with an empty vector or a plasmid expressing Flag-tagged SENP7 V5. Cells were then prepared for ChIP analysis as described above (i). An immunoblot is included to demonstrate input protein levels (ii). *n* = 3. (**F**) U2OS cells were transfected with p73–luciferase (luc) or CDC6-luciferase reporter plasmids for 48 hours, along with empty vector (vec) or Flag-tagged SENP7 V5. Reporter activity was measured, and immunoblots were performed to monitor input protein levels. *n* = 2. (**G**) Model diagram where PRMT5-mediated methylation of chromatin-associated E2F1 mediates its interaction with p100/TSN, which permits the E2F1 complex to associate with a subset of RNAs, some being derived from E2F-target genes. By regulating the activity of the splicing machinery, it is proposed that the E2F1-p100/TSN complex can influence the alternative splicing of these RNAs. In the absence of E2F1 methylation (either under conditions of PRMT5 inhibitor treatment or in cells expressing E2F1-meR point mutants), a p100/TSN-dependent interaction with the splicing machinery is lost, and changes to alternative splicing of a subset of RNAs result.

We wanted to understand the functional significance of alternative splicing directed by meR-E2F1 and p100/TSN for the E2F pathway. To this end, we decided to pursue *SENP7* as previous studies had highlighted the role of SENP7 deSUMOylase in the control of HP1, an established repressor of E2F transcriptional activity (*24*, *25*) and a known target for deSUMOylation by *SENP7* (*20*). We assessed whether the *SENP7* V5 RNA variant, which selectively interacts with p100/TSN-E2F1 and is dependent on PRMT5 activity, can influence E2F activity. We did this by measuring HP1α and SUMO ChIP activity on the *p73* promoter, an established E2F target gene (*26*). Treating cells with EPZ015666 (which down-regulates the *SENP7* V5 RNA variant; Fig. 4D) caused an increase in chromatin-associated SUMOylation on the *p73* promoter, which coincided with reduced levels of transcription (Fig. 6A and fig. S4, F and G). Moreover, the increased chromatin SUMOylation reflected an increased association of HP1α (Fig. 6B). Mechanistically, silencing SENP7 with siRNA caused increased levels of chromatin-associated HP1α (Fig. 6C); a similar effect was observed upon silencing E2F1 (Fig. 6D), thus connecting chromatin SUMOylation to E2F1 activity. We subsequently addressed the specific role of the meR-E2F1–associated spliced RNA variant by expressing *SENP7* V5 in cells and measuring the effect on HP1α ChIP activity. Expressing the V5 variant, and the resulting SENP7 protein, decreased the level of HP1α ChIP activity (Fig. 6E). Most significantly, the reduced HP1α ChIP activity coincided with increased transcriptional activity of E2F target genes (Fig. 6F). These results suggest that the V5 variant, derived from an E2F1-dependent alternative splicing effect on *SENP7*, has a functional consequence on the E2F pathway.

## DISCUSSION

The work described here provides new mechanistic insights into the processes affected by arginine methylation of E2F1 and relates the information to the fundamental properties of the E2F pathway. We found that the methylation mark not only affects the repertoire of genes transcriptionally regulated by E2F1, but most importantly, enables E2F1 to exert control over alternative RNA splicing of a large group of E2F target genes that otherwise are poor E2F transcription targets. We suggest, therefore, that the methylation mark extends the regulatory impact of E2F1 on gene expression, from one where transcriptional control is the principal level of control to another where alternative RNA splicing is the predominate process. This pathway provides a mechanism whereby E2F1 can extend its influence to genes, which otherwise would be poor transcription targets for E2F1. The meR mark thus widens the genomic landscape under E2F1 control.

We found that components of the splicing machinery associate with E2F1 and that a diverse array of RNAs, mostly derived from E2F target genes, are subject to alternative splicing control in an E2F1-dependent fashion. Moreover, by reading the symR mark on E2F1, p100/TSN recruits an extensive group of RNAs to E2F1, many of which represent alternatively spliced variants. It is known that p100/TSN functions in small nuclear ribonucleoprotein assembly and, hence, is involved with pre-mRNA splicing (*27*), and it is consistent with this observation we identified that snRNAs associated with E2F1 were dependent on PRMT5 activity and E2F1 methylation. This highlights a possible mechanism whereby E2F1 can engage with the splicing machinery to influence the splicing process (Fig. 6G).

Our results make the interesting suggestion that there is a broad division of E2F target genes into two groups: one group regulated through the classical E2F pathway mechanism of transcriptional control and the other consisting of genes that are generally poor E2F transcription targets, where regulation occurs principally through alternative RNA splicing. Reflecting on the biological properties of E2F1, we reason that this broad division into two mechanisms for controlling gene expression could have biological significance in mediating the outcome of E2F1 activity. This is because alternative RNA splicing provides the cell with a great deal of flexibility in protein function and thus may be relevant in physiological situations where the transcriptional role of E2F1 is compromised.

The analysis of alternative RNA splicing of the *SENP7* gene supports the importance of alternative splicing for E2F1 function. Thus, manipulating the expression level of V5 (the *SENP7* RNA variant dependent on PRMT5 and E2F1 activity identified in the E2F1 RIP-seq) found that it was an efficient regulator of E2F target gene transcription, most likely through altering the repressive effect of HP1α on E2F target gene activity. It appears, therefore, that the ability of E2F1 to affect alternative RNA splicing has significant functional consequences on E2F1 activity.

In conclusion, our study has revealed an unexpected mechanism whereby arginine methylation widens the regulatory impact of E2F1, from its classical mechanism of transcriptional control to one where alternative RNA splicing is the predominate level of regulation. The reader-writer interplay, which is dependent on the meR mark, endows E2F1 with a new regulatory RNA splicing mechanism that extends its genomic influence. The meR mark thus expands the repertoire of genomic landscape under E2F control.

## MATERIALS AND METHODS

### Cell line generation

Hemagglutinin (HA)–tagged WT, the arginine to lysine 111/113 mutant E2F1 (KK), and the arginine to lysine 109 (R109K) constructs have been described previously (*11*). These were subcloned into a pTRE2-hyg expression vector (Clontech) and transfected into parental Tet-On U2OS cells (Clontech; RRID: CVCL_V335) to generate inducible, stable cell lines. These cells were selected in Dulbecco’s modified Eagle medium (DMEM) (Sigma-Aldrich) supplemented with 10% (v/v) fetal bovine serum (FBS), penicillin/streptomycin, G418 (100 μg/ml; Santa Cruz Biotechnology), and hygromycin B (150 μg/ml; TOKU-E). For all experiments, doxycycline (1 μg/ml) was used to induce protein expression for 24 hours before harvest. E2F1 and TSN CRISPR cells were generated as per the protocol described (*28*) and cultured in DMEM containing 10% (v/v) FBS and penicillin/streptomycin. All cell lines were tested for mycoplasma contamination before use.

### Plasmid/siRNA transfection

HA-tagged WT E2F1, E2F1-KK, and E2F1 R109K plasmids have been described previously (*11*). HA-tagged E2F1 L132E and R166H constructs were generated from WT HA-E2F1 using a site-directed mutagenesis kit (Stratagene). Flag-tagged SENP7 V5 was generated by subcloning from an open reading frame shuttle clone (0CAAo5051G027D; Source BioScience) using primers targeting the start and stop codons (flanked with NotI and SalI restriction sites, respectively). The polymerase chain reaction (PCR) product was purified using a PCR purification kit (Qiagen) and digested with the required enzymes (Promega) for 1 hour. The digested DNA was gel-purified using a gel extraction kit (Qiagen) and ligated into the p3xFlag-CMV-7.1 vector (Sigma-Aldrich). Plasmid transfections were performed for 48 hours using the GeneJuice transfection reagent (Novagen), as per the manufacturer’s instructions. RNA interference was performed with 25 nM siRNA for 72 hours using the Oligofectamine transfection reagent (Invitrogen), as per the manufacturer’s instructions. Sequences for siRNA are as follows: nontargeting control, 5′-AGCUGACCCUGAAGUUCUU-3′; E2F1, 5′-CUCCUCGCAGAUCGUCAUCUU-3′; p100/TSN, 5′-AAGGAGCGAUCUGCUAGCUAC-3′; SENP7, 5′-GAAGUAAGACAGUAGAUGA-3′.

### Immunoblotting and antibodies

For immunoblots, cells were harvested in radioimmunoprecipitation assay buffer [50 mM tris-HCl (pH 8), 150 mM NaCl, 1% (w/v) Igepal CA-630, 0.5% (w/v) sodium deoxycholate, 0.1% (w/v) SDS, 0.2 mM sodium orthovanadate, and protease inhibitor cocktails] and incubated on ice for 30 min before SDS–polyacrylamide gel electrophoresis. The following antibodies were used in immunoblots: p100/TSN (A302-883A, Bethyl Laboratories; RRID: AB_10631268), E2F1 (C20, Santa Cruz Biotechnology; RRID: AB_631394), E2F1 (A300-766A, Bethyl Laboratories; RRID: AB_2096774), HA (16B12, Covance; RRID: AB_10063630), FLAG (M2, Sigma-Aldrich; RRID: AB_262044), β-actin (AC-74, Sigma-Aldrich; RRID: AB_476697), H4R3me2s (ab5823, Abcam; RRID: AB_10562795), histone H4 (ab10158, Abcam; RRID: AB_296888), and SENP7 (donated by R. Hay, University of Dundee, UK).

### RNA isolation and quantitative PCR

RNA was isolated from cells using TRIzol (Thermo Fisher Scientific) according to the manufacturer’s instructions. One microgram of total RNA was used for complementary DNA (cDNA) synthesis. For standard mRNA analysis, oligo(dT)_20_ (Invitrogen) was added. For splice variant analysis, RNA was deoxyribonuclease (DNase)–treated (Sigma-Aldrich) before cDNA synthesis using random hexamers (Invitrogen). Moloney Murine Leukemia virus (M-MLV) reverse transcriptase (Promega) was used as per the manufacturer’s instructions. Quantitative reverse transcription PCR (qRT-PCR) was carried out in triplicate using the indicated primer pairs and the Brilliant III SYBR Green qPCR Master Mix (Stratagene) on an MX3005P (Agilent) qPCR instrument. Results were expressed as average (mean) fold change compared to control treatments using the ΔΔ*C*_t_ method from three biological repeat samples. Glyceraldehyde-phosphate dehydrogenase or actin primer sets were used as an internal calibrator. Error bars represent SE unless otherwise indicated.

### Chromatin immunoprecipitation

ChIP was performed as described previously (*29*, *30*). Antibodies used for immunoprecipitation were as follows: anti-E2F1 (C-20), anti-HA (16B12), anti-HP1α (NB110-40623, Novus Biologicals; RRID: AB_714949), anti-SUMO2/3 (8A2, Abcam; RRID: AB_1658424), and nonspecific rabbit or mouse immunoglobulin G (IgG). The recovered DNA was analyzed in triplicate by qPCR, as described (*30*, *31*), on an MX3005P qPCR system using the Brilliant III SYBR Green qPCR Master Mix according to the manufacturer’s instructions. Results were expressed as average (mean) fold change compared to IgG control treatments using the ΔΔ*C*_t_ method from triplicate biological repeat samples. Alternatively, a standard curve was generated to calculate ChIP/input signals that were subsequently used to generate fold change values compared to IgG control. Error bars represent SE unless otherwise indicated.

### RNA immunoprecipitation

Cells were washed once with phosphate-buffered saline (PBS) before ultraviolet cross-linking at 900 mJ/cm^2^ using a Stratalinker (Stratagene). RIP lysis buffer [50 mM tris-HCl (pH 8.0), 150 mM NaCl, 1 mM MgCl_2_, 10% (v/v) glycerol, 1% (v/v) NP-40, 1 mM dithiothreitol, 0.2 mM sodium orthovanadate, and protease inhibitor cocktails] was added directly to the plate, on ice. The lysate was agitated at 4°C for 10 min before sample clarification at 13,000 rpm. For protein samples, 5% of inputs were taken and boiled in an SDS-loading buffer. For RNA samples, 10% of inputs were taken, and 10 μg of proteinase K was added for 30 min at 37°C before addition of TRIzol and RNA isolation. The rest of the lysate was precleared using preblocked protein A/G agarose beads, 1 μg of nonspecific IgG (Jackson ImmunoResearch), and heparin (0.1 mg/ml) for 1 hour at 4°C. The precleared lysate was added to a fresh tube with 1 μg of nonspecific IgG or a specific antibody (E2F1; C-20, Santa Cruz Biotechnology; RRID: AB_631394) for 1 hour with rotation. Protein A/G beads were then added for a further hour. The beads were washed four times in RIP lysis buffer and resuspended in 400 μl of RIP lysis buffer. This was separated into two fractions—one for protein isolation and the other for RNA extraction. For protein isolation, beads were dried and resuspended in SDS-loading buffer before boiling. For RNA extraction, an equal amount of RIP extraction buffer [350 mM NaCl, 10 mM tris-HCl (pH 7.5), 10 mM EDTA, 0.1% (w/v) SDS, and 7 M urea] was added to the fraction, along with 15 μg of proteinase K, and incubated at 37°C for 30 min before RNA purification using TRIzol. RNA was DNase-treated before first-strand cDNA synthesis using random hexamers and M-MLV reverse transcriptase.

### RNA sequencing

WT, E2F1-KK, or E2F1-R109K expression was induced in U2OS-Tet-ON cells for 24 hours before isolating the RNA using TRIzol. mRNA was subsequently enriched from three biological replicates using the NEBNext Poly(A) mRNA Magnetic Isolation Module (New England Biolabs), as per the manufacturer’s instructions. cDNA libraries were made using the NEBNext Ultra Directional RNA Library Prep Kit for Illumina (New England Biolabs). Sequencing was carried out on an Illumina NextSeq platform.

### RNA-seq data analysis

FASTQ files for pTRE, WT, KK, and R109K samples in three biological replicates were trimmed to remove adapters and low-quality bases with TrimGalore v.0.4.3 (www.bioinformatics.babraham.ac.uk/projects/trim_galore/). The trimmed reads were aligned to the human reference genome (build hg19) with STAR aligner v.2.5.1 (*32*) with two mismatches allowed. Differential gene expression analysis was performed with DESeq2 R Bioconductor package v.1.16.1 (*33*) using read counts data provided by the aligner. Genes were considered differentially expressed if the adjusted *P* value, calculated using the Benjamini-Hochberg method to minimize the false discovery rate (FDR), was less than 0.01, and the change in expression level was greater than twofold. Differential splicing analysis, Ψ calculation, and splicing events statistics were performed with rMATS turbo package v4.0.1 (*17*). The FDR threshold for differential percent spliced in PSI was chosen to be 0.01. The GO enrichment analysis was performed with MetaCore software suite (Clarivate Analytics, v.6.33-69110) to reveal biological processes overrepresented in differentially spliced gene sets. *P* values for GO enrichment analysis were calculated using the formula for hypergeometric distribution, reflecting the probability for a GO term to arise by chance. Statistically enriched terms were identified using a threshold FDR of 3%. Clustering of GO:BP terms was performed using the R Bioconductor goseq package (v.1.30), and annotations were provided in org.Hs.eg.db (v.3.5.0) and GO.db (v.3.5) packages. Gene expression data have been deposited in the National Center for Biotechnology Information’s (NCBI) Gene Expression Omnibus (GEO) and are accessible through GEO Series accession number GSE111961.

### RIP sequencing

An E2F1 RIP was performed as described above, from samples treated for 72 hours with nontargeting siRNA or siRNA against TSN. An E2F1 siRNA condition was also included for the RIP-seq as a control to monitor for specificity of the RNA species identified. Following RNA extraction and DNase treatment, equal volumes of the sample were taken and underwent ribodepletion using a GeneRead rRNA Depletion kit (Qiagen). Libraries were prepared using a NEBNext Ultra Directional RNA library Prep kit for Illumina (New England Biolabs). The library was sequenced on an Illumina NextSeq, and bioinformatics analysis was carried out (see below).

### RIP-seq data analysis

FASTQ files for two biological replicates in each condition were trimmed as described above. The reads were aligned to the human genome build hg19 by gsnap aligner v.2017-04-21 with two mismatches allowed (*34*). The RIP-seq analysis was performed with RIPSeeker R package v.1.18.0 (*35*) with the parameters as follows: uniqueHit = TRUE, assignMultihits = TRUE, rerunWithDisambiguatedMultihits = TRUE, and automatic bin size selection. Ensembl BioMart build 75 was used for functional annotation of the RIP-seq results. RNA species significantly enriched (adjusted *P* value threshold < 0.05) above the siE2F1 control RIP are recorded in table S4. RIP sequencing data have been deposited in NCBI’s GEO and are accessible through GEO Series accession number GSE111961.

### Gene set analysis

Gene set analysis was performed with the piano R package (v.1.8.2) using the Mean method (*36*), with 1000 permutations and with minimum and maximum gene sets of 15 and 500, respectively, against the 50 hallmark (h) gene sets from the MSigDB (v.6.1). Resulting gene sets with a nominal *P* value of 0.05 were considered significant. Distinct directional network maps were visualized with the piano R package.

### Xena browser functional genomics analysis

For the analysis of E2F1, PRMT5, MECOM V7, and total MECOM expression levels in human cancers, Xena browser (University of California) was used (https://xena.ucsc.edu/). The TCGA TARGET GTEx dataset was selected, which contained transcript expression data from TCGA (cancer tissue) and Genotype-Tissue Expression (GTEx; healthy tissue) samples. Cervical, colon, and ovarian cancers were selected alongside their respective healthy tissue and were categorized according to their E2F1 gene expression. Information on PRMT5 and MECOM gene expression was also displayed. MECOM V7 transcript was identified using the Ensembl transcript ID.

### Immunofluorescence

U2OS cells (HTB-96, American Type Culture Collection; RRID: CVCL_0042) were plated on coverslips and transfected for 48 hours with the indicated plasmids, or U2OS-Tet-ON cells were induced to express WT E2F1, E2F1-KK, or E2F1-R109K for 24 hours as appropriate. Cells were fixed for 15 min with 4% paraformaldehyde in PBS and permeabilized for 15 min with 0.5% Triton X-100 in PBS. Coverslips were incubated with primary antibody for 1 hour, washed five times, and then incubated with Alexa Fluor 488–conjugated secondary antibody (Thermo Fisher Scientific; RRID: AB_141607) for 1 hour. Coverslips were washed again before mounting on glass slides using VECTASHIELD mounting medium with DAPI (4′,6-diamidino-2-phenylindole; Vectorlabs). Proteins were visualized on a BX60 fluorescence microscope (Olympus) fitted with a Hamamatsu C4742-95 camera and analyzed with Openlab 5 software (Improvision).

### Flow cytometry

WT, E2F1-KK, or E2F1-R109K mutant U2OS-Tet-ON cells were induced with doxycycline for 24 hours before addition of fresh medium containing 20 μM etoposide and doxycycline for 48 hours. Then, cells were fixed and stained with propidium iodide for cell cycle analysis, as described previously (*30*).

### Clonogenic assay

A total of 1000 cells were seeded into six-well plates in triplicate and left to settle overnight. Doxycycline was added the following morning to induce protein expression and was topped up every 72 hours over the 10-day period. After 10 days, cells were washed twice in PBS before fixation in ice-cold methanol for 20 min. Methanol was removed, and 0.5% crystal violet stain was added for 10 min. The colonies were washed thoroughly in water and left to dry before counting.

### Luciferase reporter assays

U2OS cells were transfected with 500 ng of p73-luciferase or CDC6-luciferase plasmids, along with 500 ng of β-galactosidase and 2 μg of p3xFlag-CMV SENP7 V5 or empty vector for 48 hours. Cell extracts were then prepared in Reporter Lysis Buffer (Promega) and combined with luciferase reagent (Promega) for signal detection on a Microlumat Plus LB 96 V luminometer (Berthold Technologies). Alternatively, extract was mixed with β-galactosidase buffer [200 mM Na_2_PO_4_ (pH 7.3), 2 mM MgCl_2_, 100 mM β-mercaptoethanol, and ortho-nitrophenyl-galactosidase (1.33 mg/ml)] and incubated at 37°C before absorbance monitoring (415 nm) on a Sunrise microplate reader (Tecan). Reporter activity was determined from triplicate technical repeats as luciferase/β-galactosidase reading and expressed as fold induction compared to empty vector–expressing cells. Average (mean) fold changes with SE from two biological repeat experiments are shown.

### Statistical analysis

Statistical analyses were performed using two-tailed, unpaired Student’s *t* test with Excel software (Microsoft). Data are shown as means with SE displayed. *P* values are indicated as *P < 0.05 or **P < 0.005.

## Supplementary Material

http://advances.sciencemag.org/cgi/content/full/5/6/eaaw4640/DC1

Download PDF

Table S1

Table S2

Table S3

Table S4

Table S5

Table S6

## SUPPLEMENTARY MATERIALS

Supplementary material for this article is available at http://advances.sciencemag.org/cgi/content/full/5/6/eaaw4640/DC1 Fig. S1. Generation of stable, inducible cell lines expressing E2F1 methylation site mutants. Fig. S2. Additional analysis of RNA-seq and rMATS datasets. Fig. S3. GO biological process enrichment analysis on spliced E2F1 target genes from the RNA-seq data. Fig. S4. Additional analysis of E2F1 RIP-seq datasets. Fig. S5. Expression of E2F1 correlates with PRMT5 and MECOM V7 transcript expression in human cancer. Table S1. List of up- and down-regulated E2F1 target genes identified from the RNA-seq analysis for each cell line, corresponding to Fig. 1B. Table S2. List of alternative splicing events in E2F1 target genes identified in the RNA-seq rMATS analysis corresponding to the heatmap (Fig. 2A). Table S3. Differential expression of genes associated with RNA splicing, taken from the RNA-seq dataset (Fig. 1B). Table S4. List of RNAs identified in the anti-E2F1 RIP-seq analysis (Fig. 4). Table S5. List of overlapping E2F target genes between RIP-seq dataset (Fig. 4) and splicing analysis (Fig. 2A). Table S6. List of E2F1 RIP-seq reads that span exon junctions.
