# Supplementary material for: Arginine methylation expands the regulatory mechanisms and extends the genomic landscape under E2F control
Source: Sci Adv. 2019 Jun 26;5(6):eaaw4640. doi: 10.1126/sciadv.aaw4640 (PMC6594773; doi:10.1126/sciadv.aaw4640)
Supplement: http://advances.sciencemag.org/cgi/content/full/5/6/eaaw4640/DC1 [file supp_5_6_eaaw4640__index.html]

Science Advances | Science Advances

## Supplementary Materials

**The PDF file includes:**

- Fig. S1. Generation of stable, inducible cell lines expressing E2F1 methylation site mutants.
- Fig. S2. Additional analysis of RNA-seq and rMATS datasets.
- Fig. S3. GO biological process enrichment analysis on spliced E2F1 target genes from the RNA-seq data.
- Fig. S4. Additional analysis of E2F1 RIP-seq datasets.
- Fig. S5. Expression of E2F1 correlates with PRMT5 and MECOM V7 transcript expression in human cancer.
- Legends for tables S1 to S6

Download PDF

**Other Supplementary Material for this manuscript includes the following:**

- Table S1 (Microsoft Excel format). List of up- and down-regulated E2F1 target genes identified from the RNA-seq analysis for each cell line, corresponding to Fig. 1B.
- Table S2 (Microsoft Excel format). List of alternative splicing events in E2F1 target genes identified in the RNA-seq rMATS analysis corresponding to the heatmap (Fig. 2A).
- Table S3 (Microsoft Excel format). Differential expression of genes associated with RNA splicing, taken from the RNA-seq dataset (Fig. 1B).
- Table S4 (Microsoft Excel format). List of RNAs identified in the anti-E2F1 RIP-seq analysis (Fig. 4).
- Table S5 (Microsoft Excel format). List of overlapping E2F target genes between RIP-seq dataset (Fig. 4) and splicing analysis (Fig. 2A).
- Table S6 (Microsoft Excel format). List of E2F1 RIP-seq reads that span exon junctions.

**Files in this Data Supplement:**

- Adobe PDF - aaw4640\_SM.pdf
