# Supplementary material for: Arginine methylation expands the regulatory mechanisms and extends the genomic landscape under E2F control
Source: Sci Adv. 2019 Jun 26;5(6):eaaw4640. doi: 10.1126/sciadv.aaw4640 (PMC6594773; doi:10.1126/sciadv.aaw4640)
Supplement: Download PDF [file aaw4640_SM.pdf]

Science Advances
AAAS

advances.sciencemag.org/cgi/content/full/5/6/eaaw4640/DC1

Supplementary Materials for

Arginine methylation expands the regulatory mechanisms and extends the genomic landscape under E2F control

Alice Poppy Roworth, Simon Mark Carr, Geng Liu, Wojciech Barczak, Rebecca Louise Miller, Shonagh Munro, Alexander Kanapin, Anastasia Samsonova, Nicholas B. La Thangue*

*Corresponding author. Email: nick.lathangue@oncology.ox.ac.uk

Published 26 June 2019, *Sci. Adv.* **5**, eaaw4640 (2019)
DOI: 10.1126/sciadv.aaw4640

The PDF file includes:

Fig. S1. Generation of stable, inducible cell lines expressing E2F1 methylation site mutants.
Fig. S2. Additional analysis of RNA-seq and rMATS datasets.
Fig. S3. GO biological process enrichment analysis on spliced E2F1 target genes from the RNA-seq data.
Fig. S4. Additional analysis of E2F1 RIP-seq datasets.
Fig. S5. Expression of E2F1 correlates with PRMT5 and MECOM V7 transcript expression in human cancer.
Legends for tables S1 to S6

Other Supplementary Material for this manuscript includes the following:

(available at advances.sciencemag.org/cgi/content/full/5/6/eaaw4640/DC1)

Table S1 (Microsoft Excel format). List of up- and down-regulated E2F1 target genes identified from the RNA-seq analysis for each cell line, corresponding to Fig. 1B.
Table S2 (Microsoft Excel format). List of alternative splicing events in E2F1 target genes identified in the RNA-seq rMATS analysis corresponding to the heatmap (Fig. 2A).
Table S3 (Microsoft Excel format). Differential expression of genes associated with RNA splicing, taken from the RNA-seq dataset (Fig. 1B).
Table S4 (Microsoft Excel format). List of RNAs identified in the anti-E2F1 RIP-seq analysis (Fig. 4).
Table S5 (Microsoft Excel format). List of overlapping E2F target genes between RIP-seq dataset (Fig. 4) and splicing analysis (Fig. 2A).
Table S6 (Microsoft Excel format). List of E2F1 RIP-seq reads that span exon junctions.

Figure S1

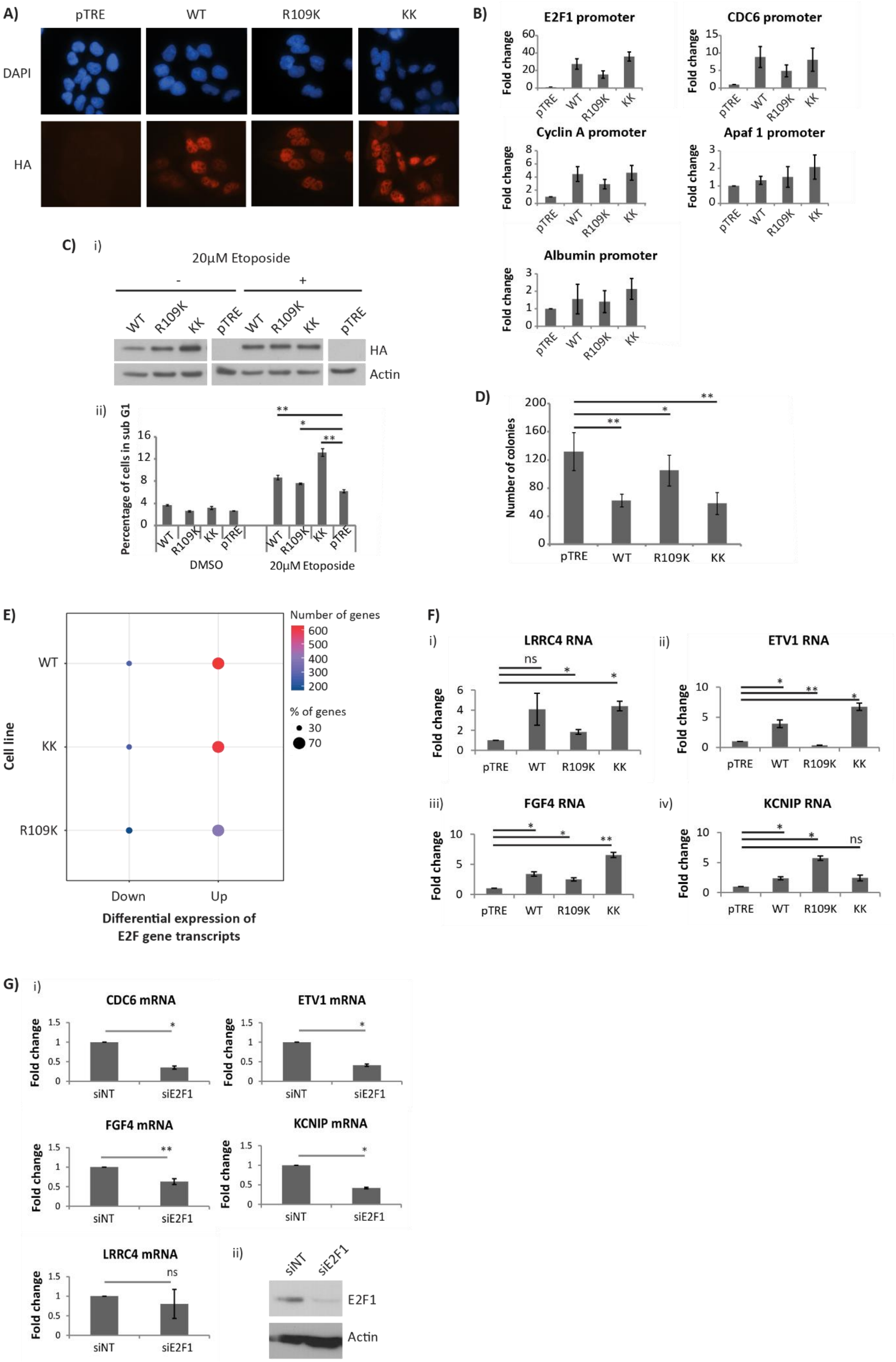
A)
pTRE
WT
R109K
KK
DAPI
HA
B)
E2F1 promoter
CDC6 promoter
Cyclin A promoter
Apaf 1 promoter
Albumin promoter
Fold change
C) i)
20µM Etoposide
HA
Actin
ii)
Percentage of cells in sub G1
DMSO
20µM Etoposide
D)
Number of colonies
E)
Number of genes
% of genes
Cell line
Down
Up
Differential expression of E2F gene transcripts
F)
LRRC4 RNA
ETV1 RNA
FGF4 RNA
KCNIP RNA
G) i)
CDC6 mRNA
ETV1 mRNA
FGF4 mRNA
KCNIP mRNA
LRRC4 mRNA
siNT
siE2F1
ii)
E2F1
Actin

Fig. S1. Generation of stable, inducible cell lines expressing E2F1 methylation site mutants. A) U2OS stable cell lines were treated with 1μg/ml doxycycline for 24h to induce expression of wild-type E2F1 (WT), E2F1 R109K, or E2F1 R111/R113K (KK) as indicated. An empty vector cell line was included as a control (pTRE). E2F1 expression and localisation was detected with an anti-HA antibody and nuclei were stained with DAPI. B) U2OS stable cell lines were treated as above to induce expression of WT E2F1, R109K, or KK. An immunoprecipitation was performed using anti-HA antibody, and isolated chromatin was analysed by QPCR using primers targeting the indicated promoters. n = 3. C) U2OS stable cell lines were treated with doxycycline as described above, then subsequently exposed to etoposide for 48h as indicated. An immunoblot was performed to demonstrate E2F1 expression (i). Cells were also prepared for flow cytometry analysis, and the average percentage of cells in sub-G1 is displayed with standard error shown (ii). n = 3. D) 1000 cells of each U2OS stable cell line was plated and treated with doxycycline to induce E2F1 protein expression for 10 days. A colony formation assay was performed and the average number of colonies per well is displayed with standard error. n = 3. E) Proportion of E2F1 target genes (containing E2F1 binding motifs in their proximal promoters [-900 to +100]) from the RNA-seq analysis on each cell line that were up- or down-regulated over 2-fold (p adj value <0.01). The size of the dot reflects a percentage of the genes and the colour corresponds to the raw numbers, as indicated in the figure. See also table S1 and fig. S2A. F) Wild-type E2F1 (WT), R109K or KK protein expression was induced in the U2OS stable cell lines by addition of 1μg/ml doxycycline for 24h. RNA was then isolated from cells and analysed by qRT-PCR using primers against target genes selected from the RNA-seq (i to iv). n = 3. G) U2OS cells were transfected with siRNA against E2F1 or non-targeting siRNA (NT) for 72h. RNA was then isolated and qRT-PCR was performed with primers targeting the indicated genes (i). An immunoblot is also included to show input protein levels (ii). n = 2.

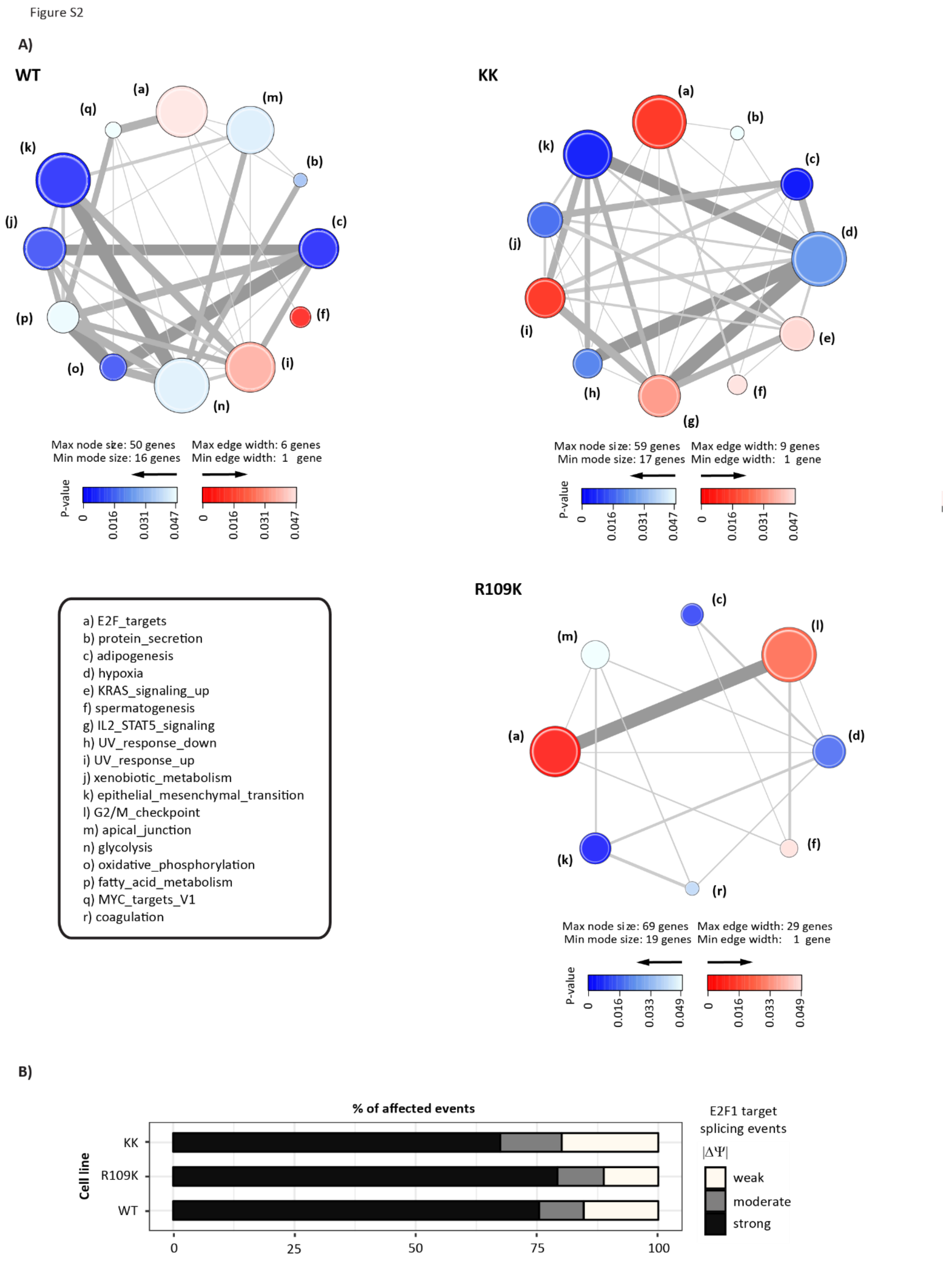

Fig. S2. Additional analysis of RNA-seq and rMATS datasets. A) Lists of upregulated and downregulated genes from each cell line (wild type [WT], R109K, R111/113K [KK]) used in the RNA-seq analysis (Fig. 1B) were utilised for GSA analysis using the piano package.

Enriched sets are represented as nodes and are coloured by significance (red indicating up-regulation and blue indicating down-regulation). Nodes are connected based on the genes they share by grey lines, with edge thickness correlating with the number of shared genes. Only differentially expressed, known E2F1 target genes with adjusted P-value < 0.01 were used in the GSA analysis. B) Magnitude of splicing changes observed in each cell line from the RNA-seq for different $\Delta\Psi$ ranges: strong (>50%), moderate (30-50%), weak (10-30%), expressed as a percentage of the total events for each cell line. This bar chart is an alternative representation of the data in Fig. 2C.

Figure S3

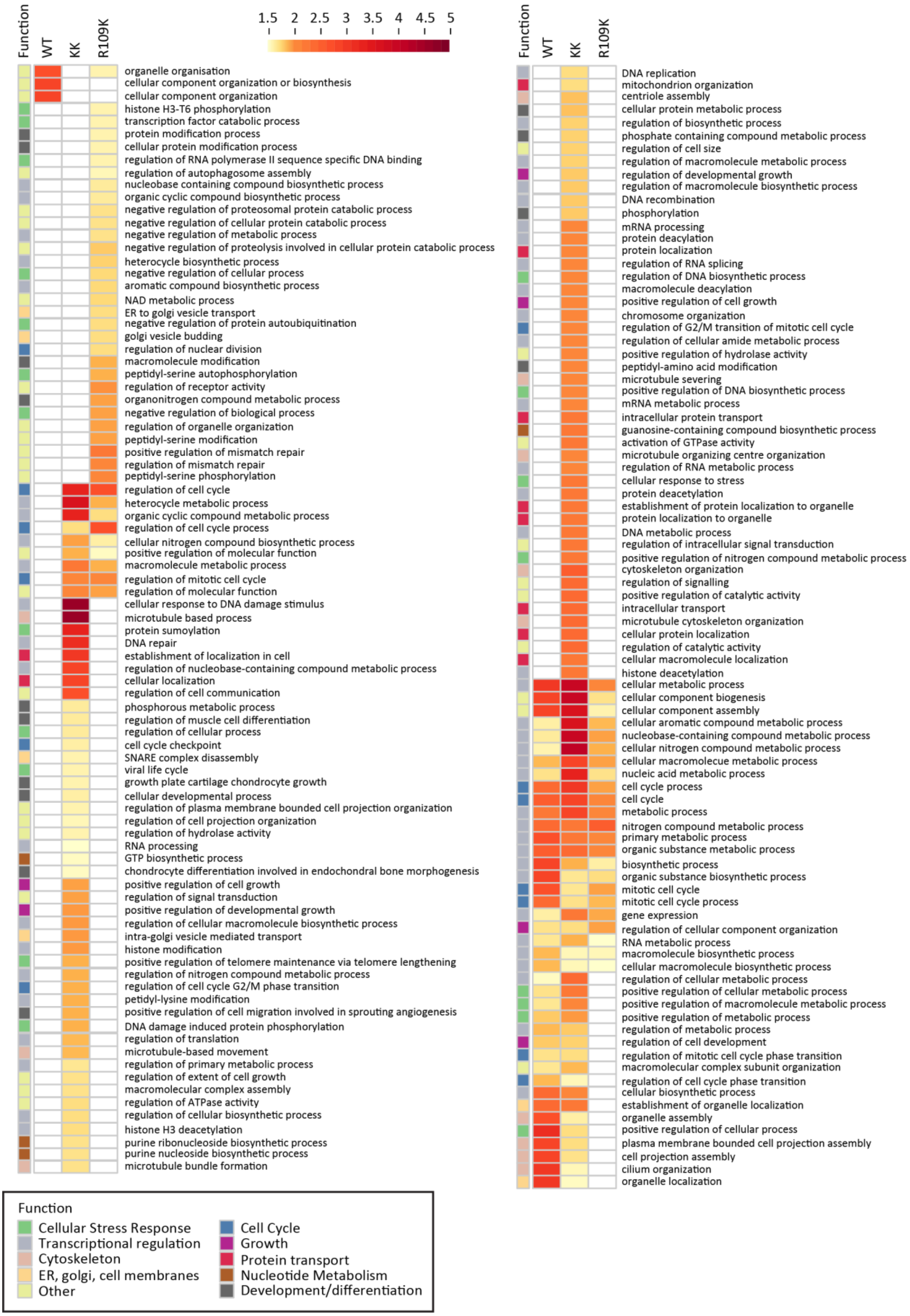
Function
WT
KK
R109K
1.5 2 2.5 3 3.5 4 4.5 5
organelle organisation
cellular component organization or biosynthesis
cellular component organization
histone H3-T6 phosphorylation
transcription factor catabolic process
protein modification process
cellular protein modification process
regulation of RNA polymerase II sequence specific DNA binding
regulation of autophagosome assembly
nucleobase containing compound biosynthetic process
organic cyclic compound biosynthetic process
negative regulation of proteosomal protein catabolic process
negative regulation of cellular protein catabolic process
negative regulation of metabolic process
negative regulation of proteolysis involved in cellular protein catabolic process
heterocycle biosynthetic process
negative regulation of cellular process
aromatic compound biosynthetic process
NAD metabolic process
ER to golgi vesicle transport
negative regulation of protein autoubiquitination
golgi vesicle budding
regulation of nuclear division
macromolecule modification
peptidyl-serine autophosphorylation
regulation of receptor activity
organonitrogen compound metabolic process
negative regulation of biological process
regulation of organelle organization
peptidyl-serine modification
positive regulation of mismatch repair
regulation of mismatch repair
peptidyl-serine phosphorylation
regulation of cell cycle
heterocycle metabolic process
organic cyclic compound metabolic process
regulation of cell cycle process
cellular nitrogen compound biosynthetic process
positive regulation of molecular function
macromolecule metabolic process
regulation of mitotic cell cycle
regulation of molecular function
cellular response to DNA damage stimulus
microtubule based process
protein sumoylation
DNA repair
establishment of localization in cell
regulation of nucleobase-containing compound metabolic process
cellular localization
regulation of cell communication
phosphorous metabolic process
regulation of muscle cell differentiation
regulation of cellular process
cell cycle checkpoint
SNARE complex disassembly
viral life cycle
growth plate cartilage chondrocyte growth
cellular developmental process
regulation of plasma membrane bounded cell projection organization
regulation of cell projection organization
regulation of hydrolase activity
RNA processing
GTP biosynthetic process
chondrocyte differentiation involved in endochondral bone morphogenesis
positive regulation of cell growth
regulation of signal transduction
positive regulation of developmental growth
regulation of cellular macromolecule biosynthetic process
intra-golgi vesicle mediated transport
histone modification
positive regulation of telomere maintenance via telomere lengthening
regulation of nitrogen compound metabolic process
regulation of cell cycle G2/M phase transition
petidyl-lysine modification
positive regulation of cell migration involved in sprouting angiogenesis
DNA damage induced protein phosphorylation
regulation of translation
microtubule-based movement
regulation of primary metabolic process
regulation of extent of cell growth
macromolecular complex assembly
regulation of ATPase activity
regulation of cellular biosynthetic process
histone H3 deacetylation
purine ribonucleoside biosynthetic process
purine nucleoside biosynthetic process
microtubule bundle formation
Function
WT
KK
R109K
DNA replication
mitochondrion organization
centriole assembly
cellular protein metabolic process
regulation of biosynthetic process
phosphate containing compound metabolic process
regulation of cell size
regulation of macromolecule metabolic process
regulation of developmental growth
regulation of macromolecule biosynthetic process
DNA recombination
phosphorylation
mRNA processing
protein deacylation
protein localization
regulation of RNA splicing
regulation of DNA biosynthetic process
macromolecule deacylation
positive regulation of cell growth
chromosome organization
regulation of G2/M transition of mitotic cell cycle
regulation of cellular amide metabolic process
positive regulation of hydrolase activity
peptidyl-amino acid modification
microtubule severing
positive regulation of DNA biosynthetic process
mRNA metabolic process
intracellular protein transport
guanosine-containing compound biosynthetic process
activation of GTPase activity
microtubule organizing centre organization
regulation of RNA metabolic process
cellular response to stress
protein deacetylation
establishment of protein localization to organelle
protein localization to organelle
DNA metabolic process
regulation of intracellular signal transduction
positive regulation of nitrogen compound metabolic process
cytoskeleton organization
regulation of signalling
positive regulation of catalytic activity
intracellular transport
microtubule cytoskeleton organization
cellular protein localization
regulation of catalytic activity
cellular macromolecule localization
histone deacetylation
cellular metabolic process
cellular component biogenesis
cellular component assembly
cellular aromatic compound metabolic process
nucleobase-containing compound metabolic process
cellular nitrogen compound metabolic process
cellular macromolecue metabolic process
nucleic acid metabolic process
cell cycle process
cell cycle
metabolic process
nitrogen compound metabolic process
primary metabolic process
organic substance metabolic process
biosynthetic process
organic substance biosynthetic process
mitotic cell cycle
mitotic cell cycle process
gene expression
regulation of cellular component organization
RNA metabolic process
macromolecule biosynthetic process
cellular macromolecule biosynthetic process
regulation of cellular metabolic process
positive regulation of cellular metabolic process
positive regulation of macromolecule metabolic process
positive regulation of metabolic process
regulation of metabolic process
regulation of cell development
regulation of mitotic cell cycle phase transition
macromolecular complex subunit organization
regulation of cell cycle phase transition
cellular biosynthetic process
establishment of organelle localization
organelle assembly
positive regulation of cellular process
plasma membrane bounded cell projection assembly
cell projection assembly
cilium organization
organelle localization
Function
Cellular Stress Response
Transcriptional regulation
Cytoskeleton
ER, golgi, cell membranes
Other
Cell Cycle
Growth
Protein transport
Nucleotide Metabolism
Development/differentiation

Fig. S3. GO biological process enrichment analysis on spliced E2F1 target genes from the RNA-seq data. GO biological process enrichment analysis was performed on the unique genes in each cell line associated with statistically significant, non-overlapping alternative splicing events (From Fig. 2A). The heatmap was generated using automatic clustering of GO:BP terms, which were then manually assigned to broader functional groupings as depicted to the bottom of the heatmap (Function).

Figure S4

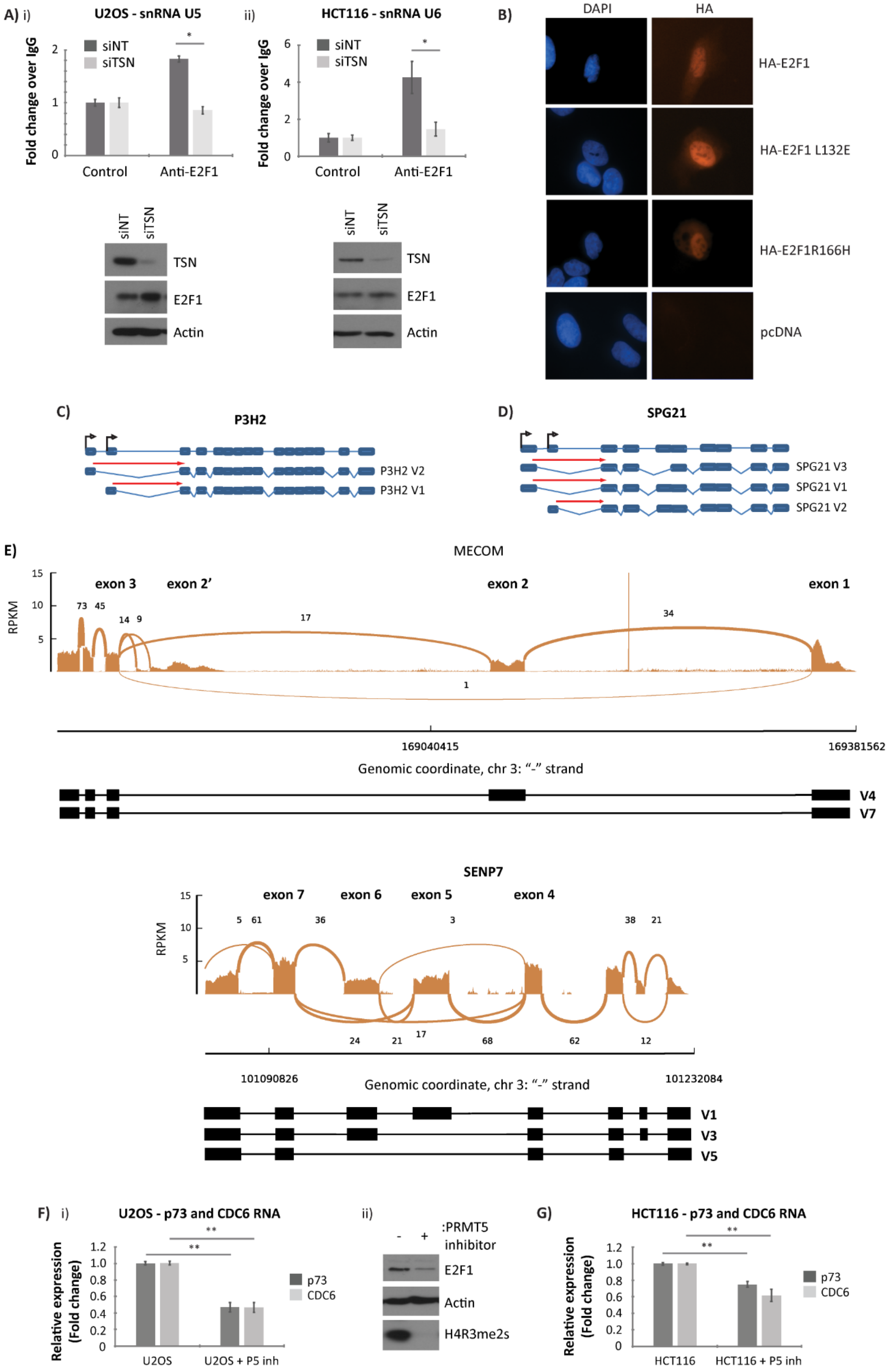
A) i)
U2OS - snRNA U5
Fold change over IgG
siNT
siTSN
Control
Anti-E2F1
ii)
HCT116 - snRNA U6
TSN
E2F1
Actin
B)
DAPI
HA
HA-E2F1
HA-E2F1 L132E
HA-E2F1R166H
pcDNA
C)
P3H2
P3H2 V2
P3H2 V1
D)
SPG21
SPG21 V3
SPG21 V1
SPG21 V2
E)
MECOM
RPKM
exon 3
exon 2'
exon 2
exon 1
169040415
169381562
Genomic coordinate, chr 3: "-" strand
V4
V7
SENP7
exon 7
exon 6
exon 5
exon 4
101090826
101232084
V1
V3
V5
F) i)
U2OS - p73 and CDC6 RNA
Relative expression (Fold change)
p73
CDC6
U2OS
U2OS + P5 inh
ii)
:PRMT5 inhibitor
E2F1
Actin
H4R3me2s
G)
HCT116 - p73 and CDC6 RNA
HCT116
HCT116 + P5 inh

Fig. S4. Additional analysis of E2F1 RIP-seq datasets. A) U2OS cells (i) and HCT116 cells (ii) were transfected for 96h with 25nM non-targeting (siNT) or p100/TSN specific siRNA, prior to lysis in RIP buffer. Cell extracts were immunoprecipitated with E2F1 antibody and co-immunoprecipitating RNA was reverse transcribed prior to QPCR analysis with primers against U5 (i) and U6 (ii) snRNAs as indicated. Input protein levels were determined by immunoblot. B) U2OS cells were transfected with HA-tagged wild-type E2F1, E2F1 L132E, E2F1 R166H, or empty vector (pcDNA) for 48h as indicated. Expression and localisation of E2F1 was detected by indirect immunofluorescence using an anti-HA antibody, whilst nuclei were stained with DAPI. C and D) Schematic diagrams representing the exon structure for each of the indicated genes: P3H2 (C), and SPG21 (D). All annotated alternative transcripts expressed from each gene are also displayed, with transcription initiation sites highlighted by black arrows. Mining of the RIP-seq data for peaks which span exon boundaries identified a number of reads that permitted specific transcript variants to be identified. These exon spanning reads are indicated on the diagrams with red arrows. E) Sashimi plots of RNA-seq data for the *MECOM* and *SENP7* gene are displayed to demonstrate that transcript variants observed in the RIP-seq (Figs. 4 and 5) could also be observed in the RNA-seq data (Fig. 2). The genomic coordinates for the highlighted regions of the gene, and a schematic of the splicing events occurring, are shown at the bottom of each graph. Exons are labelled as per the numbering system used in Fig. 4A (SENP7) and Fig. 5A (MECOM). The main panel shows the count of RNA-seq reads that span the exon junctions in this region of the gene (taken from the WT E2F1 expressing cells). F) U2OS cells were treated with 5μM PRMT5 inhibitor (P5 inh) as indicated, prior to RNA extraction and qRT-PCR analysis with primers recognising total p73 or CDC6 transcripts as indicated (i). Input protein levels are also displayed (ii). n = 4. G) As above, though the experiment was performed in HCT116 cells. Input protein levels are the same as those displayed in Fig. 4E. n = 3.

Figure S5

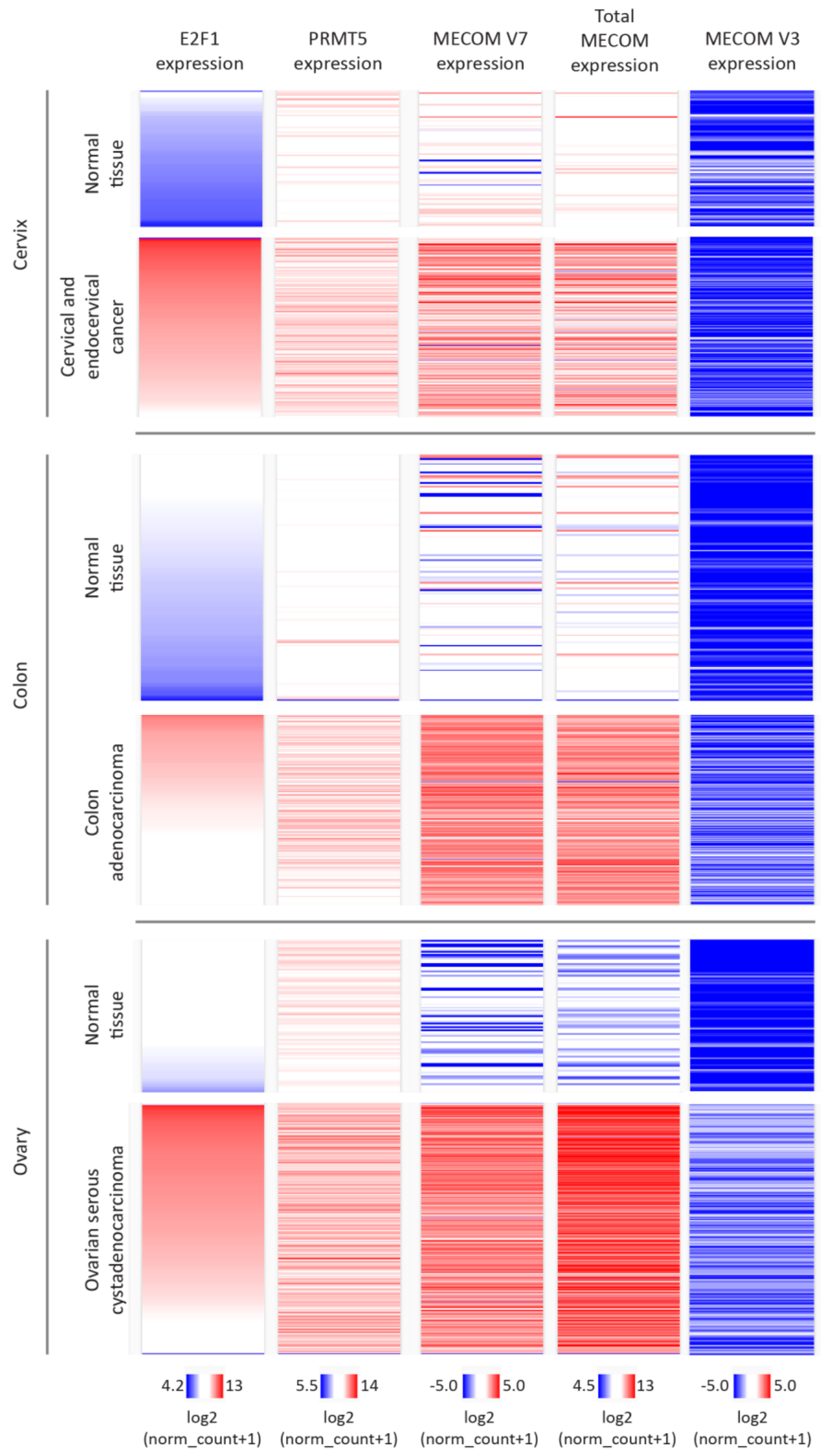

Fig. S5. Expression of E2F1 correlates with PRMT5 and MECOM V7 transcript expression in human cancer. Heatmap representation of expression levels for E2F1, PRMT5, and MECOM V7 transcripts in human cancers (cervical, colon and ovarian cancer) compared to normal tissue, generated using Xena Browser. Data from The Cancer Genome

Atlas and Genotype-Tissue Expression projects were used to display expression levels from cancer tissue or healthy tissue respectively. Each line on the heatmap represents a single patient sample, and each column represents the expression level of a particular gene (E2F1, PRMT5, MECOM) or transcript (MECOM V7). High expression levels are indicated with darker red colouring, whilst low expression levels are indicated with darker blue colouring. Cancer tissue generally displayed higher expression of E2F1 compared to normal tissue, which correlated with higher expression of PRMT5 and MECOM, particularly the MECOM V7 transcript. Other MECOM transcripts did not correlate with E2F1 expression, as exemplified by MECOM V3. FPKM; Fragments Per Kilobase of transcript per Million mapped reads.

Table S1. List of up- and down-regulated E2F1 target genes identified from the RNA-seq analysis for each cell line, corresponding to Fig. 1B.

Table S2. List of alternative splicing events in E2F1 target genes identified in the RNA-seq rMATS analysis corresponding to the heatmap (Fig. 2A).

Table S3. Differential expression of genes associated with RNA splicing, taken from the RNA-seq dataset (Fig. 1B).

Table S4. List of RNAs identified in the anti-E2F1 RIP-seq analysis (Fig. 4).

Table S5. List of overlapping E2F target genes between RIP-seq dataset (Fig. 4) and splicing analysis (Fig. 2A).

Table S6. List of E2F1 RIP-seq reads that span exon junctions.
